# First molecular characterization of *Dirofilaria* vector species and the distribution of canine dirofilariasis in Gampaha district, Sri Lanka

**DOI:** 10.3389/fcimb.2026.1775398

**Published:** 2026-04-15

**Authors:** Sajani Amarasinghe, Koshila Ranasinghe, Wasana Rodrigo, Deepika Amarasinghe, Tharaka Ranathunge

**Affiliations:** 1 Department of Zoology and Environmental Management, Faculty of Science, University of Kelaniya, Dalugama, Kelaniya, Sri Lanka; 2 Department of Zoology, Open University of Sri Lanka, Nawala, Nugegoda, Sri Lanka; 3 Department of Zoology, Faculty of Science, Eastern University, Chenkalady, Sri Lanka

**Keywords:** microfilaria, molecular-detection, mosquito, sequence, vector

## Abstract

**Background:**

Dirofilariasis is a vector-borne parasitic infection caused by filarial nematodes belonging to the genus *Dirofilaria.* Sri Lanka has reported the highest prevalence of human dirofilariasis cases in Asia. Molecular-based detection of *Dirofilaria* vector species has not yet been conducted in Sri Lanka, which could provide more sensitive information by directly detecting and analyzing genetic material. The present study aimed to analyze the distribution of canine dirofilariasis and to determine the *Dirofilaria* vector species in Gampaha district, Sri Lanka, using molecular-based techniques.

**Methods:**

Mosquito sampling (n=300) was performed from October to December 2024 from different sites in Gampaha district. The gut of the mosquitoes was dissected. Genomic DNA extraction followed by PCR was performed with specific primers for *Dirofilaria* species. The blood samples of different dog breeds (n=500) were also collected from the Gampaha district, and they were subjected to DNA extraction, PCR amplification, and sequencing. The data were analyzed by bioinformatics tools.

**Results:**

Analysis of the sequence data confirmed the first molecular detection of the dirofilariasis vector in Sri Lanka; *Armigeres subalbatus* as the potential vector mosquito for *Dirofilaria repens* and *Dirofilaria asiatica n.* sp. The sequence of *D. repens* recorded from Sri Lanka is not available in the literature/or any database, and the sequence of *D. repens* was also determined. Further, in different dog breeds, the infection rate of microfilariae was found to be different, and the Rottweiler breed was recorded with the highest infection rate. *D. repens* and *D. asiatica n.* sp. were found in dog blood samples. The phylogenetic analysis revealed that the query sequence of the *D.repens* parasites isolated from the present study showed considerable homology and proximity to the *D. repens* recorded from India.

**Conclusion:**

*Armigeres subalbatus* is the vector mosquito for *D.repens* and *D.asiatica n.* sp., which caused canine dirofilariasis in the Gampaha district of Sri Lanka. No other mosquito species were identified as potential vectors of *Dirofilaria* species in the study area. This is, to our knowledge, the first *D. repens* sequence found in the Gampaha district, Sri Lanka. The present study findings provide very important insights for targeted vector control programs for *Dirofilaria* parasites in Sri Lanka.

## Introduction

Dirofilariasis is a potentially zoonotic filarial parasitic disease and a vector-borne parasitic infection caused by filarial nematodes (McCall et al., 2008) belonging to the genus *Dirofilaria*. *Dirofilaria repens*, *Dirofilaria immitis*, *Dirofilaria tenuis*, *Dirofilaria asiatica n.* sp., and *Dirofilaria ursi* are the microorganisms that cause the disease (Boreham and Atwell, 1988; Venco et al., 2008; Ferreira et al., 2017; Genchi and Kramer, 2017; Maiti, 2022). *Dirofilaria asiatica n.* sp. denotes a newly described species within the *Dirofilaria* genus, to be formally validated as a distinct species name. In humans, dirofilariasis is often presented as pulmonary nodules (Rodrigues-Silva et al., 1995; Echeverri et al., 1999) or subcutaneous lesions (Sukumarakurup et al., 2015; Simón et al., 2017; Capelli et al., 2018; Joseph et al., 2023; Thilakarathne et al., 2023; Aththanayaka et al., 2024). In essence, dirofilariasis is a mosquito-borne (Wijetilaka et al., 1962; Genchi et al., 2009) parasitic infection that can affect both animals (Simón et al., 2012; Tereza et al., 2017; Giubega et al., 2021) and humans (Joseph et al., 2011; Roblejo-Arias et al., 2023), with varying degrees of severity (Zumaquero et al., 2020; Atapattu et al., 2023). The illness was initially documented in Sri Lanka in 1962 (Wijetilaka et al., 1962). The number of instances found has steadily increased since that time. Up to 2000, 101 cases in all had been reported from Sri Lanka (Wijetilaka et al., 1962). During that time, 91 more cases have been detected at the Department of Parasitology, Faculty of Medicine, Colombo (Wijetilaka et al., 1962). *Dirofilaria* sp. can be effectively transmitted by any mosquito species that permits microfilariae to successfully mature into the infectious L3 stage and then migrate to the proboscis (Casiraghi et al., 2006; Genchi et al., 2009; Laidoudi et al., 2021; Zinser et al., 2021; Roblejo-Arias et al., 2023). Several mosquito species in Sri Lanka can act as vectors, including *Aedes aegypti*, *Armigeres subalbatus*, *Mansonia uniformis*, and *Mansonia annulifera* (Yasuoka and Levins, 2007).

Sri Lanka has reported the highest prevalence of human dirofilariasis cases in Asia (Atapattu et al., 2023). *Dirofilaria repens* is particularly significant, causing subcutaneous dirofilariasis (Poppert et al., 2009; Simón et al., 2012; Joseph et al., 2023). In western Sri Lanka, the frequency of canine and feline *D. repens* infections keeps rising (Gunathilaka et al., 2017; Chandrasena et al., 2019; Dasanayake et al., 2022). Information on the detection of vector species responsible for Dirofilariasis in Sri Lanka is very limited. Only one study has been conducted so far, related to vector detection of filariasis in Sri Lanka (Pilagolla and Amarasinghe, 2021), doing entomological dissections only. Since organisms are prone to undergo mutations and changes in their gene sequences, there might be some changes in the initially identified species. So updated information on the distribution of the parasite species recorded within disease prevalence areas is required (Mallawarachchi et al., 2018). Compared to traditional morphological analysis, molecular diagnostic techniques offer more sensitive information by directly detecting and analyzing genetic material (Ferri et al., 2009; Soares et al., 2022; Sobotyk et al., 2022; Aththanayaka et al., 2024; Pietrzak et al., 2024). Molecular detection of *Dirofilaria* vector species has not yet been conducted in Sri Lanka. Molecular detection is a more reliable method since quantification, precise identification, and differentiation of the responsible species can be achieved (Favia et al., 1997; Casiraghi et al., 2006; Laidoudi et al., 2021; Smith et al., 2022; Aththanayaka et al., 2024).

Further, the information on the prevalence and distribution of dirofilarial parasites in the study area will help determine the potential risk of human infections, thereby taking relevant controlling measures and raising awareness (Mallawarachchi et al., 2018; Zumaquero et al., 2020). Targeted vector control measures are helpful to implement timely measures for all countries globally, especially Sri Lanka, which struggles with the financial crisis, a lack of money to buy synthetic insecticides, limited health-sector workers for control measures, and a lack of data regarding Dirofilariasis. The present study aimed to analyze the distribution of canine dirofilariasis and to determine the *Dirofilaria* vector species in Gampaha district, Sri Lanka, using molecular-based techniques to fill the knowledge gap.

## Materials and methods

### Selection of the study area

This study was conducted in the Gampaha district, which is under a red alert for filariasis. This district was selected for the study due to the available case reports, case studies, and other literature confirming a high incidence of human infection of dirofilariasis (Wijetilaka et al., 1962; Athukorala et al., 2024).

### Sampling of adult mosquitoes

Ethical clearance for conducting the study was obtained from the Institute of Biology (IOB), Sri Lanka, held on 21^st^ April 2025(Registration No: ERC IOBSL 413/03/2025).

Sampling was performed for a period of three months from October to December 2024. Mosquito sampling sites were selected from the district of Gampaha randomly, with the assistance of the Malaria Unit of Gampaha. Random sampling sites were selected, and each sampling site was geo-referenced (GARMIN-etrex SUMMIT).

Gampaha District is located in the west of Sri Lanka and has an area of 1,387 square kilometers. It is bounded by Kurunegala and Puttalam districts from the north, Kegalle District from the east, Colombo District from the south, and the Indian Ocean from the west. The climate is tropical in the Gampaha District, with significant rainfall even in the driest months. The average annual temperature in Gampaha district sites is 27.3 °C. In a year, the average rainfall is 2,000mm (Nawarathna Banda, 2016). Climatic conditions during the present study period were as follows: mean maximum daily temperature (°C) = 32.4 ± 1.4; mean minimum daily temperature = 24 ± 0.4; mean morning relative humidity (%) = 850 ± 5. 3; mean evening relative humidity = 70± 3.5; mean wind velocity (mph) = 9 ± 0.2; and total rainfall = 2,398 mm.

**Figure 1:**
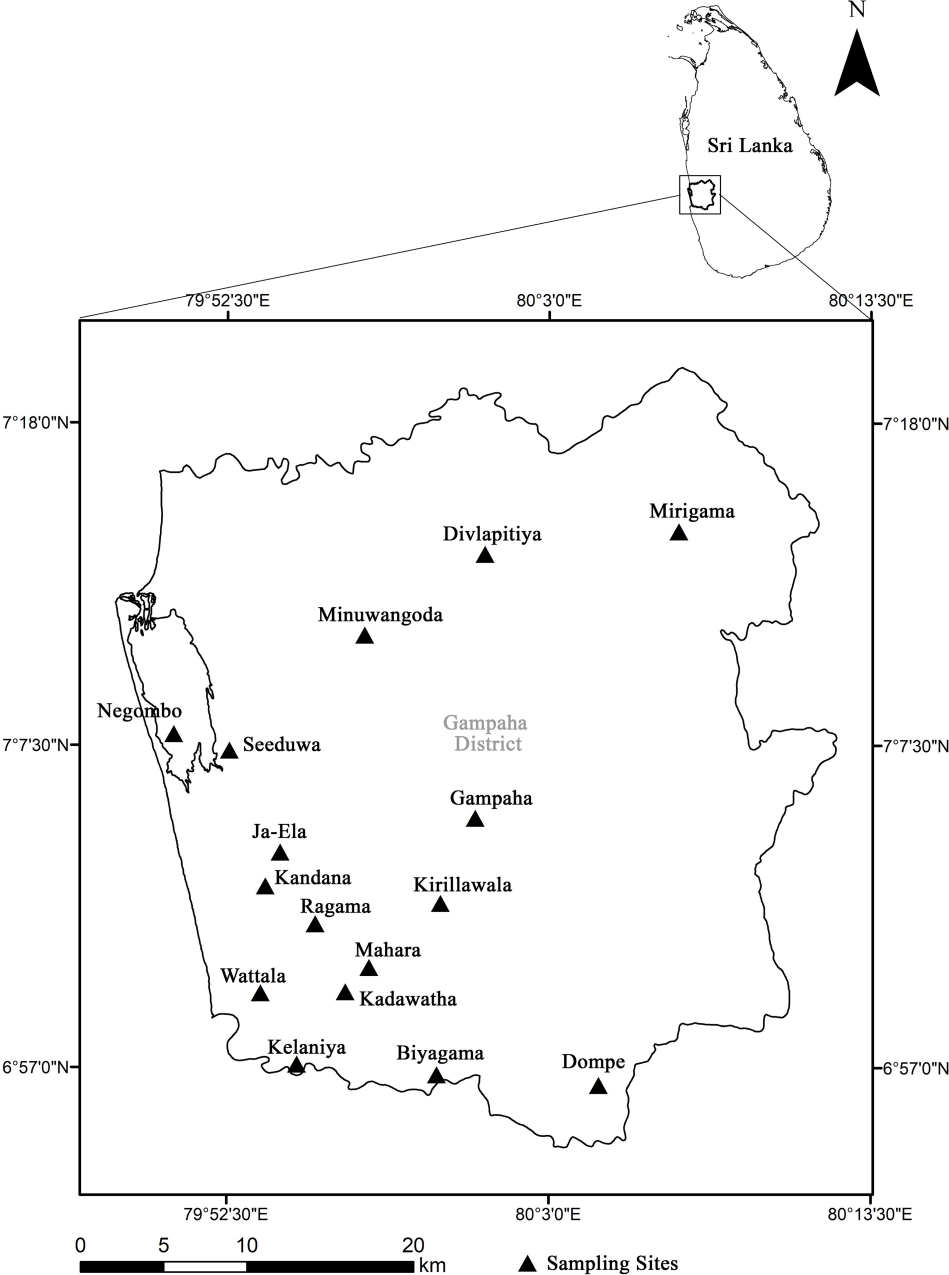
Mosquito sampling sites in Gampaha district, Sri Lanka.

Mosquito samples were collected in two-hour intervals throughout the day (From 6.00 a,m, to 7.00 p.m.) at multiple sampling sites in the Gampaha district (**Figure 1**). Sixteen areas in the Gampaha district was subjected for sampling; Divlapitiya-7.2246° N, 80.0195° E; Mirigama-7.2475° N, 80.1295° E; Minuwangoda-7.1690° N, 79.9481° E; Negombo-7.2055° N, 79.8513° E; Seeduwa-7.1247° N, 79.8750° E; Ja-Ela-7.0668° N, 79.9041° E; Gampaha-7.0915° N, 79.9948° E; Kandana-7.0478° N, 79.8970° E; Ragama-7.0280° N, 79.9230° E; Kirillawala-7.0384° N, 79.9907° E; Kadawatha-7.0046° N, 79.9542° E; Mahara-6.9909° N, 79.9396° E; Wattala-6.9907° N, 79.8932° E; Kelaniya-6.9518° N, 79.9133° E; Biyagama-6.9462° N, 79.9892° E; Dompe-6.9404° N, 80.0772° E.

Adult mosquitoes were collected by a team of 7 from the Gampaha district using 2 Prokopack aspirators and 5 mouth aspirators in all sampling locations. Collections were conducted at outdoor resting sites with a minimum distance of 5 meters between devices, following the World Health Organization guidelines (World Health Organization, 1992). Field-collected adult mosquitoes were transferred safely to an ice box. All the collected adult mosquito samples were transported to the laboratory facility at the Department of Zoology and Environmental Management, University of Kelaniya. Collected samples were immediately stored at -20 °C until further processing.

### Processing of adult mosquito samples

Mosquitoes captured from the sites were segregated within 24 hours after collection. Only good-quality captured adult mosquito samples were then subjected to identification and segregation into mosquito species based on morphological characteristics. Mosquito samples were identified at the genus and species levels using mosquito identification keys (Chelliah, 1990; Rueda et al., 2004; Rattanarithikul et al., 2005; Amerasinghe, 2009) under a low-power microscope. Only the female mosquitoes were selected for further processing. Wings and the three pairs of legs of the mosquitoes were removed. Individual mosquitoes were dissected on a glass slide using a set of dissection needles to separate the head and midgut, and salivary glands. A PBS solution was dropped onto each segment. The body segments were teased apart and cover-slipped, and the presence of any nematode parasitic stages was observed under a light microscope. Meanwhile, the head, proboscis, and thoracic tissue dissections were also cover-slipped, and the presence of any nematode larval stage parasites was observed with a compound microscope using phase-contrast optics (Olympus CX23). If any nematode stage was present, such samples were preserved in 300μL of PBS solution under sterile conditions. The dissected nematode-positive mosquito samples were mechanically homogenized by a vortex mixture (Cole-Parmer^®^ V Series Stuart Vortex Mixers) to prepare for the genomic DNA extraction.

### Genomic DNA extraction and PCR of mosquitoes for molecular screening

The total genomic DNA was extracted from 200 μL of the prepared mosquito homogenate using QIAamp DNA Mini Kit (QIAGEN Strasse 1, 40724 Hilden, Germany) following the guidelines specified by the manufacturer using 200 μL of Binding Buffer, 20 μL of Proteinase K, and 200 μL of the prepared mosquito homogenate, 500 μL of Wash Buffer 2(WB 2), 500 μL of WB 2, and 50 μL of the Elution Buffer. Then, the elute was stored at -20 °C until subjected to PCR.

PCR amplification was performed using designed primers to amplify approximately the 219 bp region of the mitochondrial cytochrome oxidase subunit I gene of *Dirofilaria* spp. parasites. Primers were designed with the sequences: Forward: DR COI-F1 (5’-AGT GTT GAT GGT CAA CCT GAA TTA-3’) and Reverse: DR COI-R1 (used one: 5’- GCC AAA ACA GGA ACA GAT AAA ACT-3’). Primers were provided in lyophilized form by AVON [AVON Pharmo Chem (PVT)LTD, NO.3, Railway Avenue, Nugegoda,10250, Sri Lanka]. The reconstitution of the primer was adopted by the protocol of Integrated DNA Technologies (IDT), following the guidelines given. A volume of the master mix mixture [Promega GoTaq^®^ Green Master Mix, Emerald Amp GT PCR Master Mix, consisting of DNA polymerase (e.g., Taq DNA polymerase), dNTPs, buffer, and MgCl_2_] was prepared that is 10% greater than the total volume required for your PCR reactions to account for pipetting errors. After preparing the master mix, specific amounts of the forward and reverse primers, template DNA, and PCR-grade water were added to reach the final volume for each reaction. The PCR was programmed for initial denaturation at 95 °C for 3 min, followed by 35 cycles of denaturation at 95 °C for 30 s, annealing at 57 °C for 30 s, and extension at 72 °C for 1 min. The final extension was at 72 °C for 1min. The amplified products were subjected to 1.5% agarose gel electrophoresis (voltage of 80V was supplied for 30–35 minutes) with Ethidium Bromide using the UV transilluminator to identify positive PCR products.

### Collection of dog blood samples & staining with Leishman stain

Blood samples were collected from several breeds (Labrador retriever, German shepherd, Rottweiler, Boxer, Stray dogs, etc.) of dogs (n=500) through the veterinarian services of pet clinics in Gampaha district, into EDTA tubes, and stored at -20 °C temperature to maintain sample integrity until testing. Then, a thin blood smear of each sample was allowed to air-dry completely and stained with Leishman stain separately to observe under a light microscope.

### Extraction of genomic DNA from dog blood

The total genomic DNA was extracted from 200 μL of the prepared dog homogenate using both QIAamp DNA Mini Kit (QIAGEN Strasse 1, 40724 Hilden, Germany) following the guidelines specified by the manufacturer and spin protocol. The DNA and protein ratios of each dog’s blood samples were measured using a Nanodrop spectrophotometer.

### DNA analysis

Specific unpurified PCR products were sent to Microgen company (Macrogen Korea 10F, 254 Beotkkot-ro, Geumcheon-gu, Seoul 08511, Rep. of Korea) through Genetech Sri Lanka (54, Kitulwatte Road, Colombo 8, Sri Lanka) to perform further characterization by sequence analysis using bidirectional Sanger sequencing. Samples that produced bands were further processed via Sanger sequencing (Eurofins Genomics, Konstanz, Germany). Microgen Company (Macrogen Korea 10F, 254 Beotkkot-ro, Geumcheon-gu, Seoul 08511, Rep. of Korea) provided results through Genetech Sri Lanka (54, Kitulwatte Road, Colombo 8, Sri Lanka).

The resulting forward and reverse sequences were analyzed using the Bio Edit tool (version 7.2) to get a final sequence by sequence alignment and analysis. Assembled full sequences were then subjected to the NCBI nucleotide BLAST tool (http://www.ncbi.nlm.nih.gov/BLAST). The search set was configured with standard database parameters, while, for the program selection, a highly similar sequence was selected. Results of the BLAST analysis, including E-value, query cover, and percent identity, were considered for choosing the sequence with ‘best hit’. Respective species were identified when their DNA sequences shared 98% -100% homology with complete DNA sequences found in the GenBank database. Evolutionary analyses were conducted using MEGA 12 software, and the phylogenetic tree was validated with the bootstrap method implemented in MEGA. Bootstrap analysis was performed with 1000 replicates to assess the statistical support for each branch in the tree. Bootstrap values, representing the percentage of replicates in which a given clade was recovered, were then assigned to the corresponding nodes in the final consensus tree. Branches with higher bootstrap values (typically ≥90%) were considered to have strong support. The evolutionary model used for tree construction and bootstrapping was Tamura-Nei, and the tree was inferred using the Neighbor-Joining method in MEGA.

## Results

### Species composition of mosquitoes in the study area

A total of 1286 adult mosquitoes belonging to 4 genera were collected from different sampling sites in Gampaha district (**Table 1**). The collection consisted of *Armigeres subalbatus* (Coquillett,1898) with the highest abundance (33.4%), followed by *Aedes albopictus* (Skuse,1895) (30.2%) and *Aedes aegypti* (Linnaeus,1762) (16.6%) (**Table 1**). Additionally, *Culex tritaeniorhynchus* (Giles, 1901) (8.71%) and *Culex gelidus* (Theobald, 1901) (8.16%) were also found in the study area. The lowest abundance in the collection was detected from *Culex fuscocephala* (Theobald, 1907) (1.17%), followed by *Mansonia annulifera* (Theobald, 1901) (1.7%).

**Table 1:** Percentage abundance of mosquito species in each sample, collected from different sampling sites of Gampaha district over several days.

Sample number	Sample sites	Captured mosquito species	Total captured number of each mosquito species.	Percentages of each captured species in each sample
Sample 1	Kadawatha Kelaniya Mahara Kirillawala	*Ae. albopictus*	82	19.4%
		*Ae. aegypti*	40	9.5%
		*M. annulifera*	22	5.2%
		*Ar. subalbatus*	252	59.6%
		*Culex* spp.	27	6.4%
Sample 2	Biyagma Dompe Gampaha Minuwangoda Mirigama	*Ae. albopictus*	144	27.4%
		*Ae. aegypti*	68	13.0%
		*Ar. subalbatus*	108	20.6%
		*Cx fuscocephala*	205	39.0%
		*Cx gelidus*		
		*Cx tritaeniorhynchus*		
Sample 3	Wattala Kelaniya Ja-Ela Seeduwa Kadana	*Ae. albopictus*	55	53.4%
		*Ae. aegypti*	28	27.2%
		*Ar. subalbatus*	20	19.4%
Sample 4	Negambo Divulapitiya	*Ae. albopictus*	108	50.0%
		*Ae. aegypti*	77	32.8%
		*Ar. subalbatus*	50	21.3%

The mosquitoes were collected as 4 samples from different areas in the Gampaha district over several days using 2 Prokopack aspirators and 5 mouth aspirators in all sampling locations. The total mosquito collections from Kadawatha, Kelaniya, Mahara, Kirillawala, Biyagma, Dompe, Gampaha, Minuwangoda, and Mirigama areas were dominated by *Ar. subalbatus* (**Table 1**). Meanwhile, the mosquito collections from Wattala, Ja-Ela, Seeduwa, and Kadana had the highest percentage abundance of *Ae. aegypti* (53.4%).

### Mosquito composition positive for the presence of nematode stages

Mosquito samples were collected from multiple locations representative of the entire study area. Five mosquito species were identified in the study area; however, only two species exhibited nematode larval-stage positivity. Only the gut contents of adult mosquitoes belonging to *Ar.subalbatus* and *Culex* spp. collected from the Gampaha district, tested positive for nematode larval stages. The highest percentage of nematode-positive samples was found in *Ar.subalbatus* (50.50%), suggesting that in the area under study, this mosquito species has more potential to carry and spread the nematode stages. The other positive samples were comprised of *Culex* spp. (22.40%). (**Table 2**). *Ae. albopictus, Ae. Aegypti* and *Mansonia* sp. were found to be negative for nematode stages (**Table 2**).

**Table 2:** Percentages of mosquito species positive for any nematode larval stages, from collected samples at the study site.

Mosquito species	Total number of mosquitoes dissected	Number of mosquitoes for the presence of nematode larval stage	Percentage (%) of mosquitoes for the presence of nematode larval stage
*Ar. subalbatus*	430	217	50.5
*Culex* spp.	232	52	22.4
*Ae. albopictus*	389	0	0
*Ae. aegypti*	213	0	0
*Mansonia* sp.	22	0	0

### Molecular detection of *Dirofilaria* species in mosquitoes

Molecular detection confirmed the presence of *Dirofilaria* species within the vector.

### Agarose gel electrophoresis images

**Figure 2:**
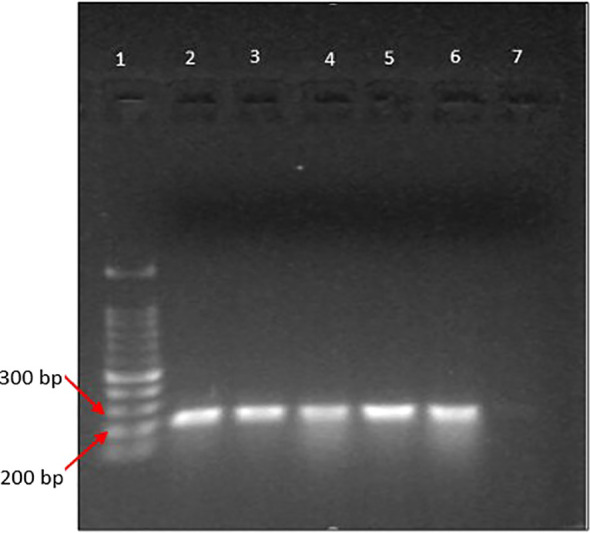
Gel image of PCR products; [lane 1: 100 bp DNA ladder (DNA ladder RTU); lane 2-6: positive for DNA of microfilariae in the gut of *Ar. subalbatus*; lane 6: negative control].

**Figure 3:**
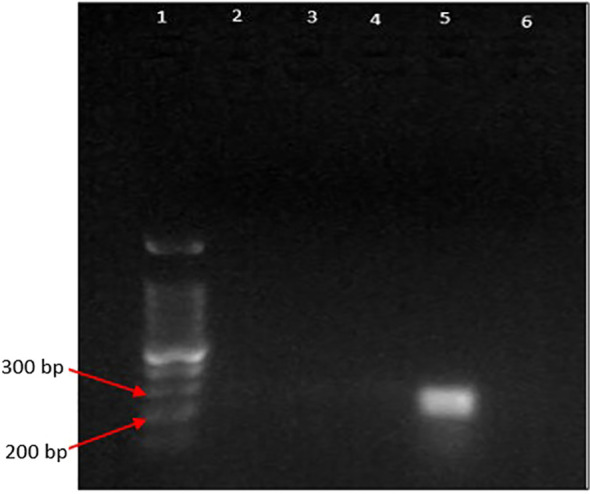
Gel image of PCR products (lane 1: 100 bp DNA ladder (DNA ladder RTU); lane 2-4: negative for DNA of microfilariae in the gut of *Culex* spp. lane 5: positive for microfilariae in the gut of *Ar. subalbatus*; lane 6: negative control).

The PCR products from the extracted DNA from gut-inhabiting nematodes in *Ar. subalbatus* were yielded with bands, approximately 200bp-300bp (**Figures 2**, **3**). The positive bands in the gel image confirm the presence of *Dirofilaria* parasites in the gut of *Ar.subalbatus* by the PCR amplification. The primer-specific PCR was run, confirming the *Ar. subalbatus* as the potential vector of *Dirofilaria* parasites. The bands in the agarose gel electrophoresis were of good quality and visible with better separation to send for sequencing.

But the PCR samples from extracted DNA of nematode worms collected from any *Culex* species were not detected with a positive band in the agarose gel electrophoresis images.

### Sequence analysis

The sequences for PCR samples were received from Macrogen, Korea, for all samples sent for sequencing. The BLAST output of the analyzed sequences was given as *D.repens*, and the relevant sample contains DNA extracted from nematode stages in *Ar. subalbatus* mosquitoes. Additionally, the BLAST output of the analyzed sequences from many samples was given as *D.asiatica n.* sp. (**Table 3**), and the relevant sample also contains DNA extracted from nematode stages in *Ar.subalbatus* mosquitoes. Although *Culex* spp. samples sometimes yielded nematode larvae on microscopy; all of these larvae were PCR-negative for *Dirofilaria*, suggesting that they likely represent other nematode species or non-confirmed *Dirofilaria* stages that do not carry detectable *Dirofilaria* DNA.

**Table 3:** BLAST output of analyzed sequences from *Dirofilaria* parasites in *Ar. subalbatus* mosquito gut.

Number of samples sent for sequencing	BLAST output
13	*Dirofilaria repens* isolate DCO1 cytochrome oxidase subunit 1 gene, partial cds; mitochondrial (Best hit with Sequence ID: KT588609.1)
14	*Dirofilaria asiatica n.* sp. isolate 207 cytochrome c oxidase subunit I (COX1) gene, partial cds; mitochondrial (Best hit with Sequence ID: PQ757307.1)

These findings confirm the first molecular detection of the dirofilariasis vector in Sri Lanka, *Ar.subalbatus* as the vector mosquito for *D.repens* and *D.asiatica n.* sp. The sequence of *D. repens* recorded from Sri Lanka is not available in the literature/or in any database, and the sequence of *D.repens* was also determined by the results.

Interestingly, for a few samples, the forward and reverse sequences failed for the sequence alignment, and the dendrograms revealed the presence of overlapping peaks with many gaps in the sequences, suggesting the presence of more than one *Dirofilaria* species in the sample.

### Identification of nematodes in dog blood samples

**Figure 4:**
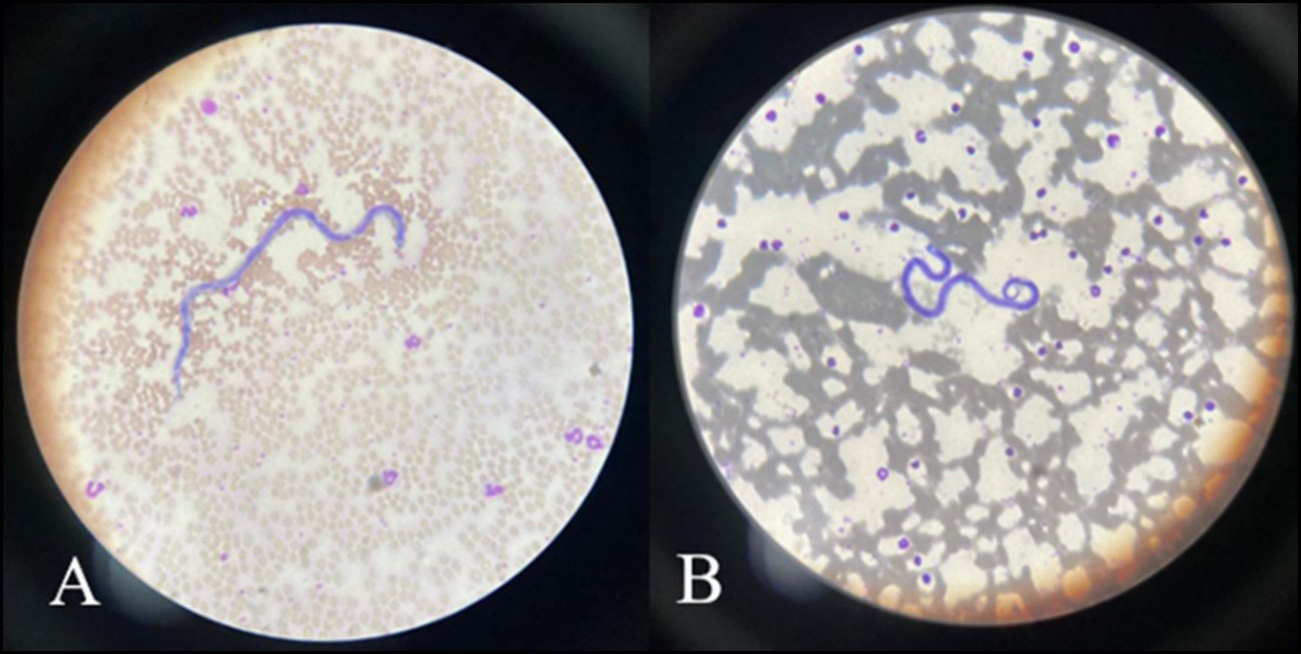
**(A, B)** Microscopic view of nematode worms in dog blood samples stained with Leishman stain (x400 magnification).

The direct blood smears stained with Leishman stain revealed the presence of nematode worms (**Figure 4**).

Nineteen positive dog blood samples were detected as positive for nematode worms from the total checked dog blood samples. The highest prevalence was observed in Labrador Retrievers, followed by crossbred dogs, with these two groups contributing 21.1% of the overall positive cases (**Table 4**). Dachshunds, stray dogs, Petit Basset Griffon Vendéen, and Boxers showed comparable susceptibility to microfilariae infection (**Table 4**).

**Table 4:** Dog breeds with percentage (%) positive for infection with Dirofilarial worms.

Dog breed and the number of dogs sampled	Positive number of samples for Infection with microfilariae	Age of the dog for positive samples (years)	Percentage (%) positive from the total number of positive samples
Cane Corso (n=45)	2	9	10.5
		8	
Labrador (n=60)	4	8	21.1
		9	
		3	
		3	
Rottweiler (n=62)	2	13	10.5
		5	
German Shepherd (n=48)	3	4	15.8
		3	
		6	
Cross breed (n=65)	4	5	21.1
		3	
		3	
		3	
Dachshund (n=50)	1	8	5.3
Stray dogs (n=65)	1	5	5.3
Petit Basset Griffon Vendeen (n=53)	1	13	5.3
Boxer (n=52)	1	9	5.3

### Molecular detection of *Dirofilaria* species in dog blood

#### DNA concentrations and DNA/Protein ratio

NanoDrop spectrophotometers confirmed the purity of proteins and nucleic acids using ultraviolet-visible (UV-Vis) absorbance for the DNA in dog blood samples (**Table 5**). DNA has high purity when the nanodrop ratio is approximately 1.8. Then all samples were within the acceptable range, confirming qualified samples for PCR.

**Table 5:** DNA concentrations and DNA/Protein ratio in DNA extractions.

Sample code	DNA concentration	DNA/Protein
A (Rottweiler)	16.5	1.84
C (Stray dog)	29.5	1.81
G (Dachshund)	31.5	1.87
H (Cross-breed)	29.7	1.98
I (Boxer)	48.5	1.83
J (German Shepherd)	33.0	1.92
K (Cross-breed)	40.9	1.87
L (Labrador)	62.3	1.91
N (Rottweiler)	189.2	0.68
Q (Cross-breed)	24.6	1.86

#### Images of agarose gel electrophoresis for PCR products of dog blood samples

**Figure 5:**
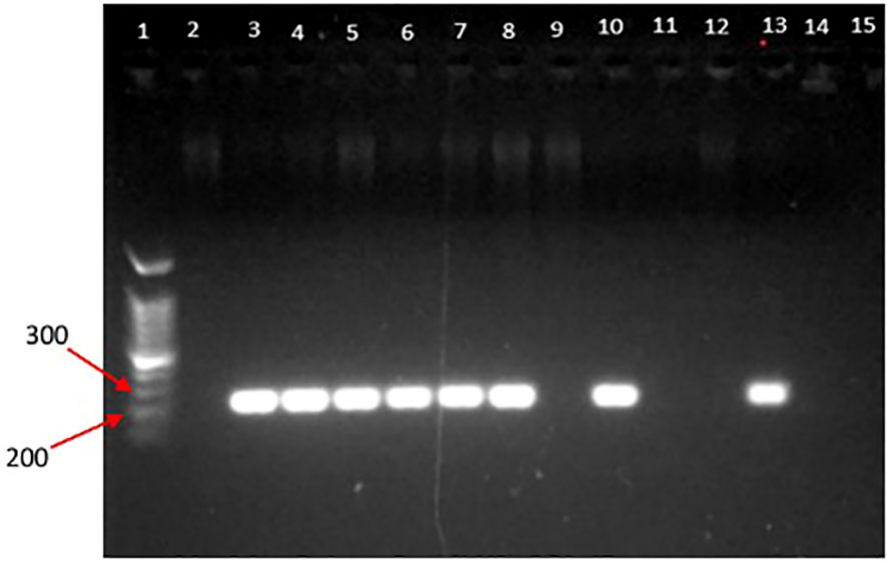
Gel image of PCR products; (lane 1: 100 bp DNA ladder (DNA ladder RTU); lane 2,9,11-12,14: negative for DNA of Dirofilarial worms in the dog blood samples.; lane 3-8, 10, 13: positive for Dirofilarial worms in the dog blood samples; lane 15: negative control).

**Figure 6:**
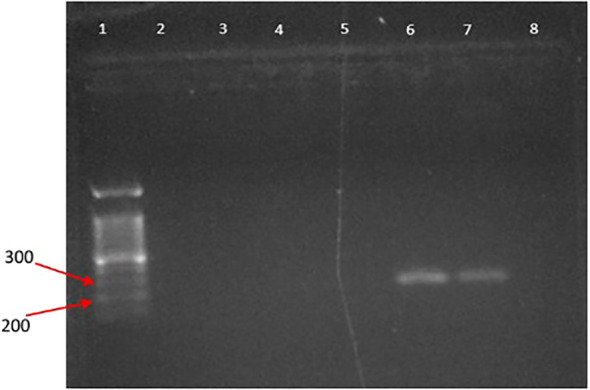
Gel image of PCR products; (lane 1: 100 bp DNA ladder (DNA ladder RTU); lane 1-5: negative for DNA of Dirofilarial worms in the dog blood samples; lane 6-7: positive for Dirofilarial worms in the dog blood samples; lane 8: negative control).

The PCR products from the extracted DNA from dog blood nematodes in different dog breeds yielded bands, approximately 200bp-300bp (**Figures 5**, **6**). The positive bands in the gel-image confirmed the presence of *Dirofilaria* parasites in the blood of several dog breeds by the PCR amplification. The bands in the agarose gel electrophoresis were of good quality and visible with better separation to send for sequencing.

But the PCR samples from extracted DNA of nematode worms loaded into lanes 2, 9, 11, 12, 14 were not detected with a positive band in the agarose gel electrophoresis image (**Figure 5**), confirming the absence of Dirofilarial worms in those samples.

#### Sequence analysis and dendrograms

Forward and reverse complement sequences were aligned with MUSCLE, and the full sequence was constructed using the Bio Edit tool for samples detected with *D. repens* (**Table 6**).

**Table 6:** Sequence analysis for sample code A1: sample detected with *D. repens* (sample sequences were submitted to the NCBI, accession no. PV436697 for the A1 sample).

Sequences type	Sequence
Forward	>H250219-010_A01_A1_COI-F.ab1 179 TAATGTTATTTTGGGATTACATACTGTAGGTATTGGTTCTTTGTTGGGTGCTATTAATTTTATAGTTACTACTCAGAATATACGTTCTACTGCTGTTACTTTAGATCAAATTAGTATGTTTGTTTGAACTTCTTATTTGACTTCTTTTCTTTTAGTTTTATCTGTTCCTGTTTTGGCTG
Reverse	>H250219-010_C01_A1_COI-R.ab1 178 GAGGAGGTCAATAAGAGTTCAACAAACATACTAATTTGATCTAAAGTAACAGCAGTAGAACGTATATTCTGAGTAGTAACTATAAAATTAATAGCACCCAACAAAGAACCAATACCTACAGTATGTAATCCCAAAATTATACTATCTAAAGATAATTCAGGTTGCCATCAACACTTGA
Complete Accession no. PV436697	>QUERY A1 CAACCTGAATTATCTTTAGATAGTATAATTTTGGGATTACATACTGTAGGTATTGGTTCTTTGTTGGGTGCTATTAATTTTATAGTTACTACTCAGAATATACGTTCTACTGCTGTTACTTTAGATCAAATTAGTATGTTTGTTTGAACTTCTTATTTGACTTCTTTTCTTTTAGTTTTATCTGTTC

#### Blast output results

The sequences for PCR samples were received from Macrogen, Korea, for all samples sent for sequencing. The BLAST output of the analyzed sequences was given as *D.repens*, confirming that the relevant samples contain DNA extracted from microfilariae in dog blood samples. The A1 sample had the best hit, D. repens isolate DCO1 cytochrome oxidase subunit 1 gene, partial cds; mitochondrial, with accession KT588609.1 (**Table 7**). The best hit (210 bp) was with a query cover of 100%, a percentage identity of 100% and an E-value of 7e-91 with zero gaps.

**Table 7:** BLAST output of analysed sequences from *Dirofilaria* parasites in dog blood samples.

Sample code sent for sequencing	BLAST output
A1 Rottweiler	*D.repens* isolate DCO1 cytochrome oxidase subunit 1 gene, partial cds; mitochondrial (Best hit with Sequence ID: KT588609.1)
A2 Stray dog	*D.asiatica n.* sp. isolate 207 cytochrome c oxidase subunit I (COX1) gene, partial cds; mitochondrial (Best hit with Sequence ID: PQ757307.1)
A5 Boxer	*D.asiatica n.* sp. isolate 207 cytochrome c oxidase subunit I (COX1) gene, partial cds; mitochondrial (Best hit with Sequence ID: PQ757307.1)
A8 Labrador	*D.repens* isolate DCO1 cytochrome oxidase subunit 1 gene, partial cds; mitochondrial (Best hit with Sequence ID: KT588609.1)

Additionally, the BLAST output of the analyzed sequences from other samples was given as *D.asiatica n.* sp. (**Table 7**), and the relevant sample also contains DNA extracted from nematode worms in dog blood samples.

Interestingly, for a few samples, the forward and reverse sequences failed for the sequence alignment, and the dendrograms revealed the presence of overlapping peaks with many gaps in the sequences, suggesting the presence of more than one *Dirofilaria* species in the sample.

#### Phylogenetic analysis of *Dirofilaria* sp. recorded from the study

**Figure 7:**
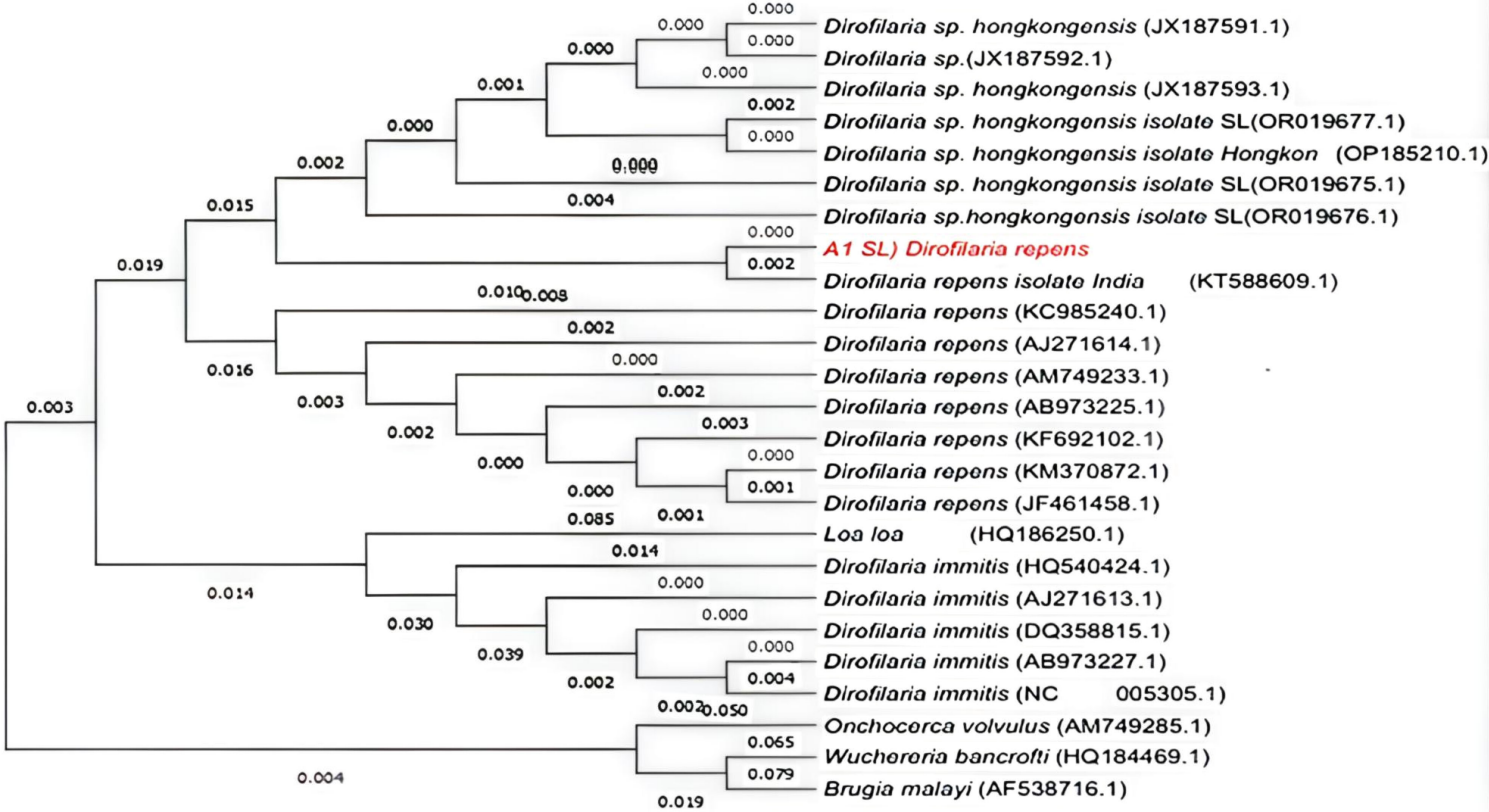
The phylogenetic relationships of the query sequence of *D. repens* with reference sequences in the biological databases.

The phylogenetic analysis revealed that the query sequence of the *D.repens* parasites isolated from the present study showed considerable homology and proximity to the sequence corresponding to a mitochondrial fragment from *Dirofilaria repens*: specifically, the cytochrome oxidase subunit 1 (COX1) gene, partial coding sequence (cds) from the DCO1 isolate, with GenBank accession KT588609.1. In GenBank terms, the LOCUS entry KT588609 (**Table 7**) describes a 210-base pair, linear DNA fragment isolated from India. The common ancestors were represented by the branch points, and genetic distances were shown by the numerical value on each branch.*D.immitis* cluster showed more divergence, and they share a recent common ancestor with comparatively small genetic variation. *O.volvulus* is clustered together, but its branch is relatively distant from the *Dirofilaria immitis*. *D.immitis* is more distantly related to *D. repens, O.volvulus*, and *D.asiatica n.* sp. *Loa loa* has a moderate degree of genetic divergence, with a distance of 0.085. With a genetic distance of 0.079, *Wuchereria bancrofti* and *Brugia malayi* are sister taxa, indicating that they are very closely related (**Figure 7**).

## Discussion

Sri Lanka has reported the highest prevalence of human dirofilariasis cases in Asia. Molecular-based detection of *Dirofilaria* vector species has not yet been conducted in Sri Lanka, which could provide more sensitive information by directly detecting and analyzing genetic material.

A previous study conducted recently had provided the first comprehensive epidemiological data on filarioids infecting pet dogs in Sri Lanka, utilizing a combination of parasitological, molecular, phylogenetic, and statistical techniques (Atapattu et al., 2023). That study reported a high prevalence of zoonotic filarial infections in dogs and identified the causative agents as *Dirofilaria* sp. ‘hongkongensis’ and *Brugia* sp. Sri Lanka genotype (Atapattu et al., 2023). The present study aimed to detect the vector of Dirofilariasis using molecular techniques for the first time in the country, following the identification of Dirofilaria species recorded from the selected study area. The previous study had used the band size in agarose gel electrophoresis to detect the species of Dirofilaria recorded, but the present study had used sequencing to confirm the presence of different Dirofilaria species.

**Figure 8:**
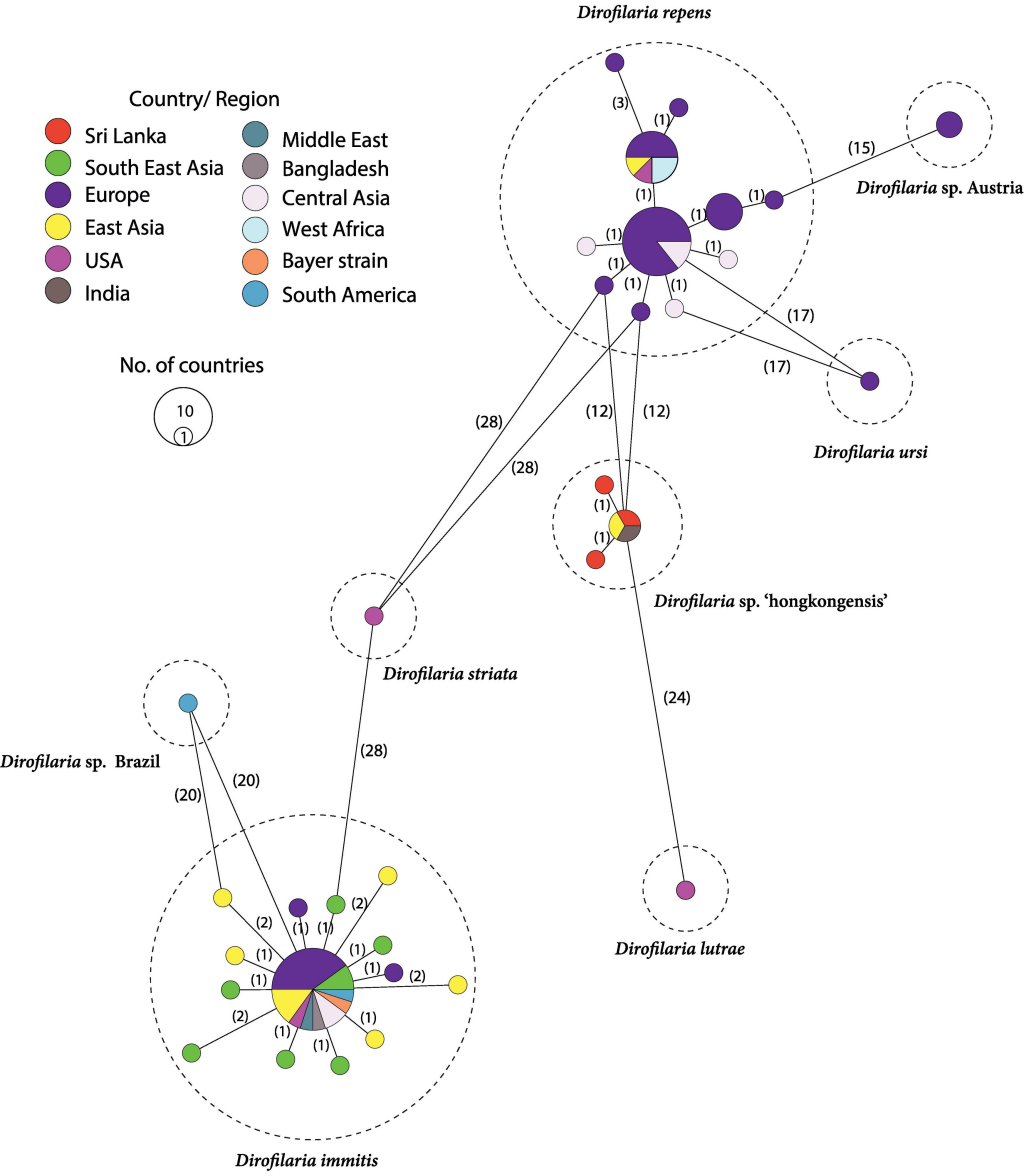
The geographic distribution of the several *Dirofilaria* sequence types found in the GenBank database, which shows both intra- and inter-specific mutations and genetic diversity of the mitochondrial cox-1 gene, was predicted from the minimum spanning networks.

According to the latest research on dogs as reservoir hosts of the zoonotic *D.asiatica n.* sp. and potentially of *Brugia* sp. Sri Lanka genotype in Sri Lanka (2023), *Dirofilaria* sequence types that are available in the GenBank database, and their geographical distribution have indicated intra- and inter-specific mutations/genetic diversity across the mitochondrial cox-1 gene (Atapattu et al., 2023) (**Figure 8**). That gene was inferred according to the minimum spanning networks. *D.asiatica n.* sp. was previously termed *Dirofilaria* sp. *‘hongkongensis’*, and due to the latest classification, it has recently been formally described and renamed *D.asiatica n.* sp (Colella et al., 2025).

In this study, we report data detailing the first investigation on vector and the distribution of canine dirofilariasis in Gampaha district, Sri Lanka through parasitological, molecular, and phylogenetic. Molecular methods were fundamental in demonstrating the vector of the zoonotic *D.asiatica n.* sp. and *D.repens* Sri Lanka genotype. Over half of the captured mosquitoes were *Ar.subalbatus*, and they were infected with *D.repens*, *D.asiatica n.* sp., accounting for the highest number of total infections, placing this parasite as the main *Dirofilaria* species infecting dogs in Sri Lanka. During our study, we were able to detect not only *D.asiatica n.* sp. but also *D.repens* in both canine blood and mosquito gut content. The results indicate that *Ar.subulbatus* facilitates the potential transmission of *D.repens* and *D.asiatica n.* sp. from one canine to another or from one animal to another. The present understanding of the epidemiology of Dirofilarial infections in Sri Lanka is hindered by a lack of information on vector detection, parasite species identification, and definitive host association with mammals. Previous findings have reported the occurrence of *D. immitis* and *D. repens* (Mallawarachchi et al., 2018; Balendran et al., 2022; Dasanayake et al., 2022; Rathnayake et al., 2022; Atapattu et al., 2023) from dogs in Sri Lanka. Only one case of *D. immitis* was found in an imported dog (Atapattu et al., 2023; Thilakarathne et al., 2023).

Human subcutaneous dirofilariasis has been prevalent nationwide since its initial identification in humans in 1962 (Wijetilaka et al., 1962), with Sri Lanka documenting the second-highest incidence globally (Rodrigues-Silva et al., 1995; Atapattu et al., 2023). Since all cases detected have been accomplished using passive surveillance, the total number of cases recorded in Sri Lanka is probably an underestimate of the true infection rate. Although *D. repens* is the main cause of human subcutaneous dirofilariasis in Europe and Central Asia, including Russia (Gardiner et al., 1978; Pampiglione and Rivasi, 1991; Settnes and Engebjerg, 1991; Muro et al., 1999; Pampiglione and Rivasi, 2000; Genchi et al., 2011; Simón et al., 2012; Kondrashin et al., 2020; Atapattu et al., 2023), new research indicates that in some regions of Asia, *D.asiatica n.* sp. may actually be the cause of most human subcutaneous *Dirofilaria* infections (Winkler et al., 2017; Pradeep et al., 2019; Kumar et al., 2021; Soares et al., 2022; Atapattu et al., 2023).

During the present study, notable differences in the species composition of mosquitoes were observed among different sampling locations. This implies that the distribution of mosquito populations may be influenced by local environmental factors, such as the availability of breeding sites and habitat features. The total mosquito collections from Kadawatha, Kelaniya, Mahara, Kirillawala, Biyagma, Dompe, Gampaha, Minuwangoda, and Mirigama areas were dominated by *Ar. Subalbatus*. Meanwhile, the mosquito collections from Wattala, Ja-El, Seeduwa, and Kadana had the highest percentage abundance of *Ae.aegypti* suggests the potential for arboviral infections.

There are ∼70 mosquito species that potentially vector *D. immitis*, with *Aedes, Anopheles*, and *Culex* as some of the most common genera (Ludlam et al., 1970; Yasuoka and Levins, 2007). Various studies in Europe have reported several species of mosquitoes infected by *D. immitis* larvae, such as (*Cx. Pipiens* (Linnaeus, 1758) in Spain, Italy, and Turkey; *Cx. theileri* in Madeira, Portugal, and on the Canary Islands, Spain; *Ae. vexans*(Meigen, 1830) in Turkey and *Ae. albopictus, Ae. caspius*(Pallas, 1771), *An. Maculipennis*(Meigen, 1818), and *Cq. richiardii* (Ficalbi, 1889) in Italy (Nayar and Knight, 1999).

Furthermore, twelve different mosquito species *[Aedes albopictus, Aedes sollicitans* (Walker, 1856), *Aedes sticticus* (Meigen, 1838*), Aedes taeniorhynus* (Wiedemann, 1821)*, Aedes trivitattus* (Coquillett, 1902)*, Aedes vexans* (Meigen, 1830)*, Anopheles bradleyi* (King, 1939) *Anopheles crucians* (Wiedemann, 1828)*, Anopheles punctipennis (*Say, 1823)*, Culex nigripalpis* (Theobald, 1901)*, Culex quinquefasciatus* (Say, 1823) *and Culex salinarius* (Coquillett, 1904)*]* are found in the southeastern United States and have been implicated as presumptive vectors of *D. immitis* (Licitra et al., 2010). *Aedes albopictus* is an invasive heartworm vector from Asia (Nayar and Knight, 1999; Licitra et al., 2010) that blood-feeds aggressively on a wide range of hosts (Cancrini et al., 2003; Bell, 2016).

During the dog blood sampling through pet clinics, blood samples found to be positive for microfilariae under the thick blood smear test were processed for DNA extraction. Usually, veterinarians examine for the presence of microfilariae in dogs that are older than 2 1/2 years. Older dogs are more vulnerable to dirofilariasis. During the blood sample collection, a range of ages was represented from each breed, ensuring that a diverse range of dogs were sampled.

According to our study findings, dirofilariasis prevalence may be influenced by age. Anyhow, breed-specific differences also exist in the percentage of dogs found positive for microfilariae. This might point to possible variations in the condition’s susceptibility among breeds. Larger sample numbers and more thorough research are necessary to reach firm findings, nevertheless. The observed trends may be influenced by environmental factors such as pet management techniques, vector prevalence, and climate (Nath et al., 2013). To have a more thorough understanding, future research could be performed to examine possible risk factors (such as lifestyle choices and preventative actions).

Molecular detection by PCR and sequencing was used as an accurate detection of the vector with high accuracy (Cheng et al., 2023), and it is a valuable diagnostic tool rather than the morphological identification (Ferri et al., 2009; Sobotyk et al., 2022; Aththanayaka et al., 2024) of the filarial nematode since quantification, precise identification, and differentiation of the responsible species is also feasible with molecular detection (Ferri et al., 2009). PCR diagnostic methods, which are based on amplifying and sequencing DNA, are very specific and can explain the difference between parasites of the same genus, such as *D.repens* and *D.immitis*, with close evolutionary relationships (Manfredi et al., 2001; Rattanarithikul et al., 2005). During the present study, to create precise clones of the target DNA exponentially, these processes are repeated (or “cycled”) 35 times. The number of cycles was optimized during the study to obtain a sufficient quantity of DNA to be sent for sequencing (Favia et al., 1996; Silbermayr et al., 2014).

The conditions optimized during DNA extraction and PCR during the present study have proven to be an accurate, reliable, and sensitive way to find very small amounts of genomic DNA coded for mitochondrial gene cytochrome c oxidase subunit 1 (cox1) of nematode parasites present in mosquito gut and blood of dogs. The PCR reaction conditions, sequence accuracy, and amplification yield and specificity are the main causes of common PCR problems. Low or no amplification, non-specific amplification or smear, sequence error with PCR products, and false positive amplification are a few of such common issues (Favia et al., 1996). Excess DNA input, poor integrity, complex sequences (e.g., GC-rich or secondary structures), long targets, problematic design and high quantity of primers, excess DNA polymerase, inappropriate DNA polymerase, excess Mg^2+^ concentration, insufficient denaturation, increased denaturation, incorrect annealing temperature, low annealing temperature, long annealing time, high extension temperature, insufficient extension time, high number of PCR cycles are some of the courses for nonspecific amplification or smears (Vakalis et al., 1999).

According to BLAST results of the present study, the best hit sequence was selected considering the hit that yields the lowest E-value, the highest query cover, and the highest percentage identity. However, overlapping peaks were observed for the dendrograms received from the sequencing of a few samples. They displayed more than one set of peaks that overlap. The presence of several *Dirofilaria* species in the same sample could be the cause. The BLAST results confirmed that prediction as well. The best hit sequence did not give the species name, although it did match *Dirofilaria* sp. as the best hit. Therefore, next-level research is recommended using *Dirofilaria* species-specific primers based on the findings of the present study.

The study findings revealed the evolutionary link of *D. repens* identified from Sri Lanka. That gave the highest similarity with *D. repens* isolated from India (DCO1 cytochrome oxidase subunit 1 gene, partial cds; mitochondrial, with accession KT588609.1) rather than *D. repens* recorded from other Asian countries. Understanding these patterns and spotting evolutionary tendencies are common goals for researchers. Based on the findings of this study, the presence of *Dirofilaria* parasites showed a significant distribution in the study area. Therefore, there is a clear indication of the immediate need for the implementation of control and preventive strategies for the prevention of rising cases of human infection with dirofilariasis.

The composition of mosquito species varied across sites, influenced by environmental factors. Even though more research studies are needed to explain the factors responsible for the attraction of vectors to diverse breeding sites, many researchers have identified breeding habitat availability as the key factor that determines the preference and composition (Herath et al., 2024). Water quality parameters in breeding environments play a significant role in egg hatching and the growth of the progeny from larvae to adults. Because of this, gravid female mosquitoes are sensitive to both biotic and abiotic factors such as organic matter, bacteria, phosphate, ammonia, and potassium content of the water during the breeding exercise. These factors are known to be closely related to the abundance of larvae and adults in the field. Although the presence of heavy metals such as iron, zinc, and copper has been found at various concentrations in breeding sites, their relationship with larval development remains unknown (Herath et al., 2024). Mosquito communities in urban and rural landscapes are generally characterized by a lower mosquito diversity and/or abundance than in natural areas. Although marshlands and other temporary flooded areas are generally absent from urban areas, human activity creates artificial habitats such as water deposits, swimming pools, urban sewage systems, gardens, and subterranean and stormwater systems that act as alternative breeding sites for mosquitoes (Ferraguti et al., 2016). Blood feeding is a core mosquito behavior with implications for pathogen transmission and control. This behavior can be examined from two perspectives: patterns and preferences. Feeding patterns are evaluated through blood meal analyses, which reveal mosquito–host associations shaped by environmental and biological factors (Fikrig and Harrington, 2021). The risk of mosquito-borne disease transmission to humans hinges largely on how much female mosquitoes feed on humans, which is itself affected by the abundance and distribution of other potential hosts. Regarding biting rhythms, risk varies with the peak biting times. Exophilic species active during the evening crepuscular period could potentially infect humans during the first 1–2 hours after sunset, when people are still outdoors in the rural farming community where this study was conducted. Species that bite later at night would encounter human hosts primarily inside dwellings, so vector–host contact would be largely determined by the level of endophily of the local mosquito population (Amerasinghe and Indrajith, 1995).

The biting cycle of *Armigeres subalbatus* is distinctly crepuscular, with two activity peaks: a smaller peak at dawn and a larger peak at dusk. The cycle is entrained to natural light-dark rhythms, with a 24-hour interval between successive dawn-to-dawn or dusk-to-dusk peaks and approximately a 12-hour interval between dawn-to-dusk or dusk-to-dawn peaks, measured at 50% activity (Pandian and Chandrashekaran, 1980). This rhythm persists day after day without notable qualitative changes. The rate of change in light intensity likely triggers the onset of crepuscular biting, as the abrupt rise (to approximately 17 lux) or fall (to about 4 lux) in ambient light at sunrise or sunset coincides with peak biting activity (Pandian and Chandrashekaran, 1980).

Host-seeking female density fluctuates with lunar phases, rising during the full moon and falling during the new moon. Although outdoor populations exceed indoor populations at ground level and on the first floor, peak biting activity occurs simultaneously across indoor and outdoor sites. A vertical stratification of biting activity was also observed (Pandian and Chandrashekaran, 1980).

Out of many measures that can be taken to control human infections of dirofilariasis, identification of the vector has been fruitfully achieved by the current study. There are several regular measures that can be taken against mosquito bites, such as using mosquito repellents, using mosquito nets, and destroying the breeding habitats of the potential vector (Pilagolla and Amarasinghe, 2021). But the importance of adhering to these measures should be further highlighted in order to prevent the alarming number of cases. Among all these measures, one significant measure that can be taken to halt the alarmingly high number of cases is increasing the awareness of human infection of dirofilariasis among the general public. All these measures can therefore be used to prevent and reduce the human infection of dirofilariasis effectively. Based on the findings of the present study, some PCR samples revealed the presence of overlapping peaks, samples showing overlapping chromatogram peaks and gaps suggest possible co−infection. But this cannot definitively resolve the species composition with the short cox1 fragment; species−specific PCR or longer sequencing would be needed to confirm these mixed infections. Furthermore, integrating veterinary surveillance, based on the bionomics and behavioral characteristics of the *Armigeres subalbatus* vector management, and public awareness programs is recommended to prevent human infection of dirofilariasis. Veterinary Surveillances helps to establish systematic monitoring of animal populations to detect early signs of disease, enabling timely interventions. Implement targeted vector control measures, such as habitat reduction, use of insecticides, and biological control agents, within the country to reduce vector populations. These integrated strategies ensure a comprehensive response that addresses multiple factors of disease transmission and promotes sustainable prevention in Sri Lanka.

## Conclusions

The present study findings confirm the first evidence on molecular detection of the dirofilariasis vector in Sri Lanka. *Armigeres subalbatus*, identified as the potential vector mosquito for *D.repens* and *D. asiatica n.* sp. detected in Gampaha district, Sri Lanka, and other districts in Sri Lanka were not evaluated in this study. No other mosquito species were identified as potential vectors of *Dirofilaria* species in the study area. This is, to our knowledge, the first D. repens cox1 sequence from Sri Lanka submitted to GenBank (accession PV436697). Sequence analysis results revealed *D.repens* and *D. asiatica n.* sp. as the *Dirofilaria p*arasites infecting dogs in the study area. The phylogenetic analysis revealed that the query sequence of the *D.repens* parasites isolated from the present study showed considerable homology and proximity to the *D.repens* isolate DCO1 cytochrome oxidase subunit 1 gene, partial cds; mitochondrial isolated from India (Sequence ID: KT588609.1).

## Data Availability

The datasets presented in this study can be found in online repositories. The names of the repository/repositories and accession number(s) can be found below: https://www.ncbi.nlm.nih.gov/genbank/, PV436697.
